# Association of lamina cribrosa morphometry with retinal nerve fiber layer loss and visual field defects in primary open angle glaucoma

**DOI:** 10.12669/pjms.36.3.1553

**Published:** 2020

**Authors:** Ayesha Saba Naz, Aisha Qamar, Sama Ul Haque, Yawar Zaman, Faisal Faheem

**Affiliations:** 1 Dr. Ayesha Saba Naz, (MBBS, Postgraduate student Anatomy) Department of Anatomy, Bahria University Medical and Dental College, Karachi, Pakistan; 2 Prof. Dr. Aisha Qamar, (MPhil Anatomy), Department of Anatomy, Bahria University Medical and Dental College, Karachi, Pakistan; 3 Dr. Yawar Zaman, (FRCS Ophthalmology, MCPS, FCPS Vitreo- Retinal, FRCS Glasgow), Assistant Professor, Dept. of Ophthalmology, Eye Specialist Liaquat College of Medicine and Dentistry & Darul Sehat Hospital, Karachi, Pakistan; 4 Dr. Sama Ul Haque, (MPhil Anatomy) Assistant Professor, Department of Anatomy, Bahria University Medical and Dental College, Karachi, Pakistan; 5 Muhammad Faisal Faheem, (M. Sc Statistics), Researcher, Department of Physiotherapy Bahria University Medical and Dental College, Karachi, Pakistan

**Keywords:** Lamina cribrosa thickness, Lamina cribrosa depth, Visual field defects, Primary open angle glaucoma, SDOCT

## Abstract

**Objectives:**

To calculate the anterior lamina cribrosa depth (ALCD) and lamina cribrosa thickness (LCT) in primary open angle glaucoma (POAG) patients and controls and to correlate lamina cribrosa (LC) parameters to retinal nerve fiber layer thickness (RNFLT) and visual field (VF) defects.

**Methods:**

The study was conducted from November 2018 to March 2019. A total of 60 correspondents (30 cases and 30 controls) were assessed for general ophthalmological investigations including intraocular pressure (IOP), axial length AXL, ophthalmoscopy, visual field (VF) testing and spectral domain ocular computed tomography (SDOCT).

**Results:**

The mean age of subjects was 62 years (Cases 67.30±1.2, controls 57.32±1.1) with more male participants. Intraocular pressure [IOP (19.85 ±1.4)], AXL (22.85 ± 1.6), VF defects (8.30 ± 4.5), RNFLT (72.58 ± 13.2) and LCT (162.51 ± 64.62) were statistically significant in POAG patients as compared to the controls.

**Conclusion:**

A thinner LC in POAG correlated significantly with the RNFLT and VF defects. LC anatomical parameters can be estimated with precision using SDOCT with enhanced depth imaging (EDI).

## INTRODUCTION

Spectrum of glaucoma includes diverse ocular disorders. All types have in common chronic glaucomatous optic neuropathy (GON) accompanied by optic nerve head (ONH) surface anatomical derangements, neuroretinal rim (NRR) thinning and alterations of the retinal nerve fiber layer (RNFL) culminating in typical visual field defects(VF).^1^ Primary open-angle glaucoma (POAG) is one of the foremost causes of global burden of eye diseases, accounting for 4.7 million DALYs with catastrophic complication of blindness^2^, hence the importance of its early detection and prevention. Lamina cribrosa (LC) has been recognized as principal site of GON damage.^3^ Anatomical insults of LC promote RNFL defects; its distortion and posterior displacement kinks the ganglionic axons within laminar pores inculcating irreversible ischemic and neuronal loss to RNFL. LC deformations have clinically been proven to precede early RNFL thinning^4^, hence its in-vivo imaging merit significance. Quantitative evaluation of in-vivo LC linear markers; anterior LC depth (ALCD) and LC thickness (LCT) has become possible due to advent of enhanced depth imaging (EDI) spectral domain optical coherence tomography (SDOCT) and taken by storm the interest of researchers. Studies have proved glaucomatous LC to be thinner and deeper in comparison to normal eyes.^5^ Greater the magnitude of LCD and LCT, the more severe RNFL thinning and subsequent VF defects may be conjectured, therefore measurements of these novel LC markers merits clinical attention and highlight the structure-function relationship. First time in Pakistan we assessed the LCD and LCT using EDI-SDOCT and correlated the LC structure with RNFL thickness and VF defects in POAG and normal subjects which we trust can prove as a beneficial prophylactic measure against the devastating blindness caused by glaucoma.

## METHODS

This observational case-control study was conducted at Al-Ain Eye Institute, Karachi. The research protocol was approved by Institutional Review Board of Bahria University Medical and Dental College, Karachi (reference No: ERC 60/2018). Study firmly adhered to the tenets of the Declaration of Helsinki. All participants were explained the procedure of the study, an informed consent was obtained before subject enrollment and participation.

### Study Subjects

For this study, POAG cases and controls aged above 20 years (Cases 67.30±1.2, controls 57.32±1.1) were enrolled from November 2018 to March 2019. All contributors were subjected to thorough ophthalmoscopic evaluation including refraction, visual acuity, slit-lamp biomicroscopy (Topcon SL-D 7, Topcon Corporation, Tokyo, Japan), Goldmann applanation tonometry (At-900, Haag Striet, Switzerland), axial length [(AXL); Optical Biometer Al- Scan Class I device, Nidek, Gamagori, Japan], standard automated perimetry (SAP), using 50-2 glaucoma testing (Medmont M700 Automated Perimeter, fast threshold, Vermont, Australia) with detailed examination of optic disc. RNFL thickness was measured using the circular scan protocol of SDOCT.

For inclusion eyes with POAG were to have best corrected visual acuity of >20/40 and AXL of <30 mm, presence of open angle on slit-lamp, clear cornea with not more than a moderate cataract. Any optic, systemic or neurological condition affecting VF, refractive or intraocular surgeries, inability to perform the examinations were excluded from the study.^4^

### Optic Disc Findings

Protocols used for POAG were presence of an open angle on slit-lamp and ophthalmoscopy with glaucomatous optic nerve damage, NRR thinning, parapapillary atrophy (PPA), and optic nerve head hemorrhages (ONHH) in the absence of any other abnormalities that could relate to findings.^1^

### Visual Field Findings

A glaucomatous VF defect in the SAP was defined as i) outside normal limits on Glaucoma Hemifield Test; ii) 3 abnormal points with P<5% probability of being normal, 1 with P <1% by pattern deviation; or iii) a pattern standard deviation (PSD) of < 5% as confirmed on two consecutive tests. VF tests should have a false-positive, false-negative and fixation loss rates of < 25%.^1^

Healthy controls had IOP of <21 mm Hg with an absence of glaucomatous disc with intact NRR, no ONHH and PPA. A normal VF had absence of glaucomatous VF defects.^1^

### Imaging and Data Acquisition

EDI-OCT imaging, (REVO nx/SOCT Copernicus REVO OPTOPOL Technology, Wavelength 830nm, Axial resolution 2µm, scan speed 1,10,000 scans/sec, scan time 1.37seconds, OPTOPOL Technology Sp. Z o.o, ul. Zabia 42, 42-400 Zawiercie, Poland) was performed bilaterally and a series of vertical B-scans obtained. The OCT was set at 2.4mm diameter centered on optic disc, this area was scanned with approximately 128 B-scan images. Approximately 1024 SDOCT images were averaged for each section. We used three horizontal B-scans, one passing through the center, one mid-superior and one mid-inferior encompassing the ONH. Only good-quality scans with quality score ≥16 were used.

### Estimation of LCD and LCT:

LCD was measured from the reference line drawn from the two terminal points of Bruch’s membrane opening (BMO) as the deepest vertical distance from the anterior surface of LC. LCT was calculated as the distance between anterior and posterior LC surfaces. The mean values of LCD and LCT at three different points were taken. All the observations of the ophthalmoscopic history and visual field were masked from the observer XYZ. Eye with greater disc defect likelihood severity (DDLS) was chosen.^3^

**Fig.1:**
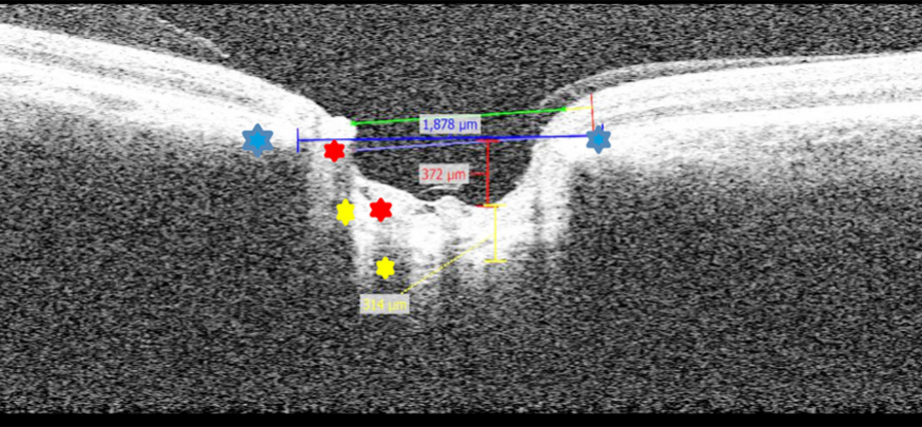
The reference line is drawn manually with blue shows Bruch’s membrane opening BMO, the red line, drawn from the BMO reference line till the deepest anterior surface ONH gives the LC Depth LCD, the yellow line drawn from the anterior opaque part of ONH to the posterior part shows the LC thickness.

### Statistical Analysis

Statistical analysis was performed using Statistical Package for Social Sciences version 23.0 for Windows (SPSS, Inc., Chicago, IL). Demographic attributes of age, gender, IOP, AXL, PSD and RNFLT were compared using chi-square test. Comparison of visual field with other study variables was inferred by independent sample t-test. Normality of data was assessed by making histogram with normal curve. Level of significance was set at P<0.05.

## RESULTS

### Baseline Characteristics

The present study included 60 eyes of 27 POAG cases and 27 eyes of healthy, age matched controls. A further 6 respondents were excluded due to tilted disc and poor image quality obscuring visualization of ALCS. No difference was found in the age or gender of the respondents whereas AXL, IOP, PSD and RNFLT were significantly higher in POAG groups as compared to the controls (Table-I).

**Table-I:** Data are mean ± standard deviation or (%) values.

	POAG (n=27)	Controls (n= 27)	P-value
Age (years)	67.30±1.2	57.32±1.1	0.939
Male	14 (51.9%)	16 (57.1%)	0.694
Female	13 (48.1%)	12 (42.9%)	
Axial length	22.85±1.6	22.0±1.1	0.04*
IOP	19.85±1.4	13.61±2.4	0.000*
PSD	8.30±4.5	3.28±1.6	0.000*
RNFLT	72.58±13.2	80.14±7.7	0.014*

Statistically significant values are presented with asterisk

* IOP: intraocular pressure,

PSD: pattern standard deviation, RNFLT: retinal nerve fiber layer thickness.

**Fig.2:**
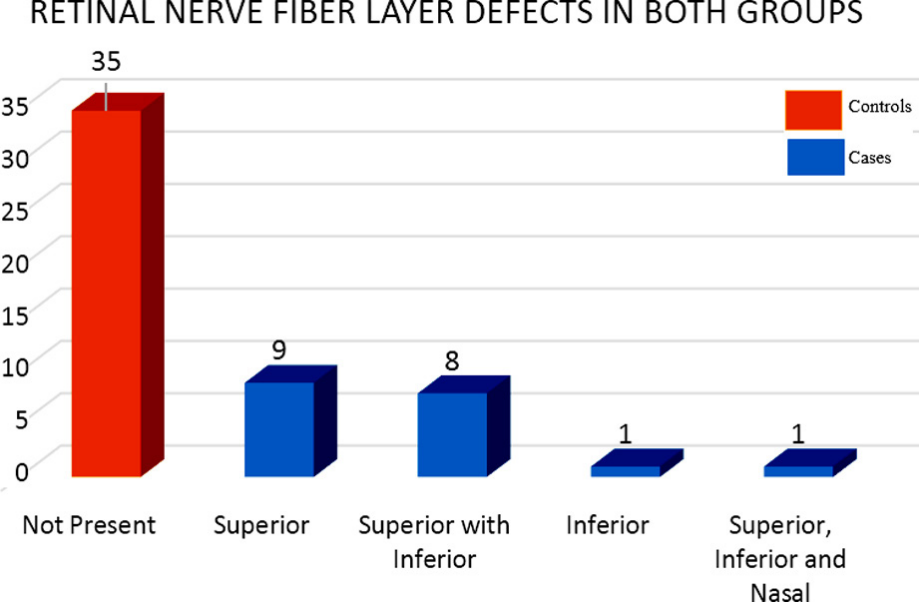
Retinal nerve fiber layer defects in superior, inferior, superior and inferior and superior, inferior and nasal sectors in POAG and controls.

### Comparison of visual field loss with general, clinical, functional and lamina cribrosa parameters

Presence or absence of visual field defects was compared with general ophthalmological, optic nerve head, outcome and LC parameters in cases and controls. Increase in the severity of IOP, AXL, vertical cup-to-disc ratio (VCDR) and PSD with decreased RNFLT and LCT produced statistically significant results in cases compared to controls.

## DISCUSSION

In this pioneer study done for the first time in Pakistan, we detected a thinner LC in POAG that corresponded well with the RNFLT and VF defects.

**Fig.3:**
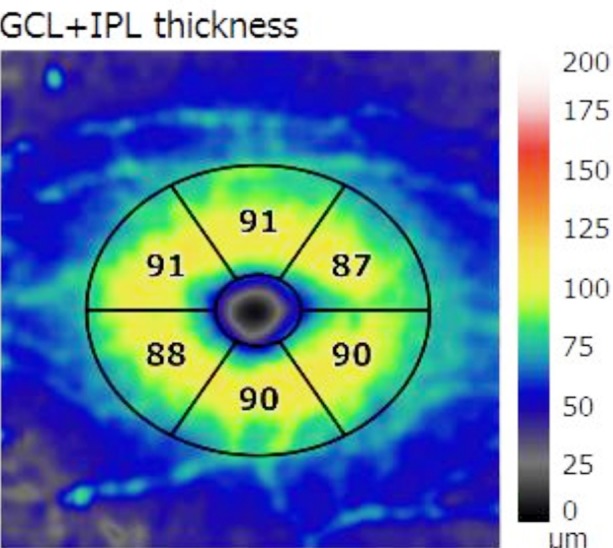
Retinal nerve fiber layer thickness in superior temporal, superior nasal, inferior temporal, inferior nasal, superior and inferior sectors (µm).

**Table-II:** Comparison of general, structural and clinical parameters between POAG cases with visual field defects and controls without visual field defects.

	Parameters	POAG with VF defects	Controls without VF defects	P- value
General Ophthalomoscopic parameters	Axial length (mm)	22.85 ± 1.07	22.03 ± 1.18	0.04*
	Intraocular pressure	19.85 ± 1.46	13.61 ± 2.50	0.000*
Functional outcome parameters	Pattern standard deviation (PSD- dB)	8.30 ± 4.56	3.28 ± 1.60	0.000*
	Retinal Nerve Fiber Layer Thickness (RNFLT-µm)	72.59 ± 13.27	80.14 ± 7.76	0.014*
Clinical optic nerve head parameters	Disc area (mm^2^)	2.45 ± 0.44	2.35 ± 0.47	0.379
	Rim Area (mm^2^)	1.01 ± 0.58	1.05 ± 0.55	0.782
	Cup Area (mm^2^)	1.44 ± 0.76	1.27 ± 0.56	0.35
	Vertical cup-to-disc ratio (VCDR)	0.95 ± 0.16	0.47 ± 0.08	0.000*
	Cup-to-disc ratio	0.60 ± 0.28	0.55 ± 0.22	0.44
Lamina cribrosa parameters	Lamina cribrosa thickness (LCT-µm)	162.51 ± 64.62	276.05 ± 74.65	0.000*
	Lamina Cribrosa Depth (LCD-µm)	312.5 ± 147.1	339.6 ± 148.3	0.500

* P-value was statistically significant at < 0.05 *P-value was statistically significant at < 0.05.

We found greater AXL of eyes corresponded well with POAG severity and LC defects. Similar significant results in AXL had also been found in a study done by Wu et al,^6^ in which the range of AXL slightly differed (21.8-30.1) in cases of POAG, but it can well be attributed to racial differences. Greater AXL were significantly associated with POAG and its severity as is also showed by Takayama et al.^7^

Currently the only modifiable parameter in management of glaucoma is IOP, hence much interest of researchers has been found in investigating this parameter. Gizzi et al^8^ explained that raised IOP can significantly produce LC anatomical changes which can either be both anterior or posterior. They had also categorized the severity of glaucoma with increments in IOP on POAG cases. The repercussions of fluctuating IOP over ONH parameters can be ascribed to LC position and compression, as described by Vianna et al,^9^ in a research fetching significant results . In our study we have also found similar statistically significant raised IOP in POAG cases and can well relate the variable to LC morphological alterations.

Recently anatomical aberrations of LC had been a great area of attention for scientists, but the authentication of it can only be proved by VF loss. Kwun et al^10^ had elaborated that lesser LC thickness can be associated better with VF defects as compared to increased thickness of LC in the controls with no loss of VF. Kim M, et al^11^ had also proved VF defects to be linked with LC cupping and thinning with glaucoma severity. The results obtained in our study also are in agreement with studies done previously. We also attained statistically significant results showing a wide range of PSD (8.30 ± 4.56), correlating well with status of glaucoma in POAG cases.

LC is a porous structure giving stability and nourishment to RGC axons, so thinning and deepening of LC could have profound implications on RNFLT and subsequent developing VF defects. In our study majority of cases were seen to have superior RNFL defects, followed by Superior- Inferior both and then inferior defects. Park et al^12^ also revealed analogous results, and attributed thinner LC with superior RNFL defects, whereas inferior ones were more allied with focal LC defects. Moghimi^13^ with his team also shared comparable conclusions of greater RNFL thinning in patients with LC defects than in controls and affected sectors also were matching our outcomes. Lee et al^14^ also stated the fact that a deeper and thinner LC is associated with a faster loss of RNFL.

Optic disc attaining vertical appearance can be attributed to loss of NRR and RGC axons. Kim YW et al had also authenticated our discovery of a significant VCDR to LC parameters in POAG.^15^ A large VCDR of ≥ 0.8 is a hallmark of GON and can rightly be associated with LC bowing.

In our study cases revealed significant LC thinning as compared to controls, and similar results have also been proposed by Hao et al^16^ in which LC thinning had been detected in POAG and primary angle closure glaucoma patients. Park et al also expounded that LC irregularities correlate with the RNFL thinning in POAG subjects.^12^ Kwun and team had correlated VF defects with a compressed LC in POAG, and results harmonized with work that we had done.^10^

### Limitations of the study

This pioneer study about LC characteristics done for the first time ever in Pakistan should had been multicentric, but unavailability of SDOCT at most medical centers made this impossible. Moreover majority of study subjects belonged to a single ethnicity. Participants should had been followed to see progression of LC parameters and glaucoma severity.

## CONCLUSION

We conclude that a thinner LC in POAG correlated significantly with the RNFLT and VF defects. LC parameters also significantly correlated with a greater AXL, raised IOP, RNFLD specifically with more predilection in superior retinal sectors and a VCDR. LC anatomical parameters can be estimated with precision using SDOCT with EDI. LC parameters can prove prophylactic against GON.

### Authors Contribution:

**ASN:** conceived, designed and wrote manuscript.

**AQ:** did review and final approval of manuscript.

**YZ:** supervised in the clinical acquisition of data.

**FF:** did the statistical analysis of the manuscript.

**SUH:** did editing of the manuscript.
